# Global spatial distribution of *Chromolaena odorata* habitat under climate change: random forest modeling of one of the 100 worst invasive alien species

**DOI:** 10.1038/s41598-023-36358-z

**Published:** 2023-06-16

**Authors:** Pradeep Adhikari, Yong Ho Lee, Anil Poudel, Sun Hee Hong, Yong-Soon Park

**Affiliations:** 1 Institute of Humanities and Ecology Consensus Resilience Lab, Hankyong National University, Anseong, 17579 Republic of Korea; 2 OJeong Resilience Institute, Korea University, Seoul, 02841 Republic of Korea; 3 School of Plant Science and Landscape Architecture, College of Agriculture and Life Sciences, Hankyong National University, Anseong, 17579 Republic of Korea; 4 Department of Plant Resources, College of Industrial Sciences, Kongju National University, Yesan, 32439 Republic of Korea; 5 Agricultural and Fisheries Life Science Research Institute, College of Industrial Sciences, Kongju National University, Yesan, 32439 Republic of Korea

**Keywords:** Climate-change ecology, Ecological modelling

## Abstract

Anthropogenic activities and global climate change increase the risk of *Chromolaena odorata* invasion and habitat expansion. To predict its global distribution and habitat suitability under climate change, a random forest (RF) model was employed. The RF model, utilizing default parameters, analyzed species presence data and background information. The model revealed that the current spatial distribution of *C. odorata* covers 7,892,447 km^2^. Predictions for 2061*–* 2080 indicate expansion of suitable habitat (42.59 and 46.30%), reduction of suitable habit (12.92 and 12.20%), and preservation of suitable habitat (87.08 and 87.80%) under the SSP (Shared Socio-economic Pathway) 2-4.5 and SSP5-8.5 scenarios, respectively, in comparison to the present distribution. Currently, *C*. *odorata* is predominantly found in South America, with limited presence in other continents. However, the data suggest that climate change will elevate the global invasion risk of *C*. *odorata* worldwide, particularly in Oceania, Africa, and Australia. Countries such as Gambia, Guinea-Bissau, and Lesotho, which currently have unsuitable habitats, are predicted to have highly suitable habitats with climate change, supporting the idea that global habitat expansion for *C. odorata* will occur due to climate change. This study indicates that proper management of *C. odorata* is crucial during the early invasion phase.

## Introduction

*Chromolaena odorata* is a perennial shrub in the Asteraceae family, indigenous to the tropical and subtropical regions of Central and South America, encompassing Mexico, the Caribbean, and Brazil^1^. *C*. *odorata* is a herbaceous to woody plant and can grow up to 2 m in height^1, 2^. It reproduces both vegetatively and sexually, with high fecundity and rapid germination^1^. *C. odorata* can grow in various types of soils, ranging from sand dunes to heavy clays, and the seeds can survive in the soil for up to 6 years^3^. *C. odorata* can tolerate acidic to neutral soil and establishes well in disturbed areas, grassland, forest, and fallow areas^1^. Because it has both short- and long-distance dispersal and can spread quickly, *C. odorata* is considered one of the world’s most invasive weeds^2, 4^.

*C*. *odorata* is recognized as a very serious weed in all types of tropical perennial crops, e.g., coffee, cocoa, and citrus^5^. *C*. *odorata* transmits pathogenic fungi^6^ and acts as a host for insect pests such as *Zonecerus variegatus*^7^, whose nymphs feed on the foliage of native plants^1^, resulting in economic loss in invaded regions.* C*. *odorata* was introduced to India in the 1800s as an ornamental plant; it spread to South East Asia by 1920^1^, West Africa by 1937, East Timor by 1975^8^, and Australia by 1994, through contaminated pasture and forest seeds^1^. It has since spread throughout Africa, Oceania, and South and South East Asia as a result of anthropogenic activities, including international trade and tourism, transportation, and land-use change, as well as natural phenomena, such as winds, tides, surface runoff, and the movement of animals^3^.

*C*. *odorata* can grow in areas with ~ 1000 mm annual rainfall to > 3000 mm annual rainfall and cold temperature from 0 to > 35 °C^1^. However, it prefers tropical climates, such as tropical rainforest, tropical monsoon, and warm temperate climates^1, 9^. Because *C*. *odorata* can survive in a wide range of environments including regions with high temperature and rainfall, it is likely to become highly abundant under future climate change, although semiarid, Mediterranean, and cold temperate climates are considered safe from invasion^9^. *C*. *odorata* can grow when days are short and bloom in the winter^10^, enhancing its ability to dominate neighboring flora and establish new habitats^11^. Thus, to prevent invasion and conserve native biodiversity, it is essential to understand how *C. odorata* will colonize, establish, and spread under climate change. Estimating the potential invasion risk of *C*. *odorata* for different regions and different climate change scenarios will enable us to focus efforts toward solid quarantine, early detection, and eradication of the species at national and international levels. Therefore, ecological niche modeling (ENM) approach has been introduced to predict alien and invasive^12^.

The ENM is widely used in the fields of ecology and conservation. The ENM combines environmental variables and species-occurrence records to predict the spatial distribution of species^13^. More recently, ENM has been extensively used to assess the geographical distribution and invasion risk of alien species in order to develop appropriate conservation strategies^12–15^. Various modeling techniques are used to fit ENMs^16^. Earlier studies mostly used regression-based models^17^, but attention has since turned toward algorithms that use machine learning (ML) techniques^18^, which have several advantages. ML techniques have more flexible fitting functions, can handle different types of data, have options to automatically select variables, and have higher predictive performance than other techniques^18, 19^. Among ENMs, random forest (RF) modeling is a novel and popular approach to ecological mapping that has high predictive performance^15^. The RF models can make sound predictions with little parameter tuning, can handle thousands of input and correlated variables; can readily assess the importance of each variable; and are robust, creating several decision trees from the majority vote^20–22^.

Our previous study determined that an RF model produced the most accurate predictions for invasive plants in South Korea among five ENMs tested (a generalized linear model, multivariate adaptive regression splines, an artificial neural network, maximum entropy, and an RF model)^15^. Most of our previous studies focused on invasive species that currently exist in South Korea^23–25^, although one study showed that future climate change will likely result in suitable habitats for a new invasive species (*Parthenium hysterophorus*)^26^. All of the studies concluded that invasive species will pose a significant threat to South Korea in the future because of climate change. Given the qualitative features of RF models, and following the recent use of ENMs to predict invasive species^14, 27^, we decided to use RF modeling to predict the spatial distribution of *C*. *odorata* at the global scale. To our knowledge, no studies have yet attempted to reveal the global invasion risk of *C*. *odorata*.

We collected global occurrence data for *C*. *odorata* and designed this study with the following main objectives: (1) to predict the current and future spatial distribution of *C*. *odorata* across the world using the RF algorithm, (2) to estimate the potential habitat change for *C*. *odorata* under future climate change, and (3) to classify the *C*. *odorata* habitat suitability in different countries of the world. The results of this study will help us to understand how the current distribution of *C. odorata* and suitable habitat will change in the future in different countries. From these results, management plans for each country (and the world) can be prepared to manage future *C*. *odorata* habitat expansion.

## Results

### Selection of bioclimatic variables and their contribution to the model

We downloaded data for 19 bioclimatic variables from the WorldClim database and performed Spearman’s correlation test (Table S1) to select the most important variables for global spatial RF modeling of *C. odorata*. Six bioclimatic variables (annual mean temperature [Bio01], mean diurnal temperature range [Bio2], isothermality [Bio03], annual precipitation [Bio12], precipitation in the wettest month [Bio13], and precipitation in the driest month [Bio 14]; Table 1) were selected on the basis of their weak correlation with each other (r < 0.75). These variables were considered to be the most significant factors for predicting the global distribution of *C. odorata*.

**Table 1:** Bioclimatic variables selected for modeling of *C*. *odorata*.

Code	Description	Unit	Model contribution (%)
Bio1	Annual mean temperature	°C	12.59
Bio2	Mean diurnal temperature range	°C	0.40
Bio3	Isothermality (Bio2/Bio7) (* 100)	%	23.75
Bio12	Annual precipitation	mm	2.40
Bio13	Precipitation in the wettest month	mm	69.58
Bio14	Precipitation in the driest month	mm	3.25

**Figure 1:**
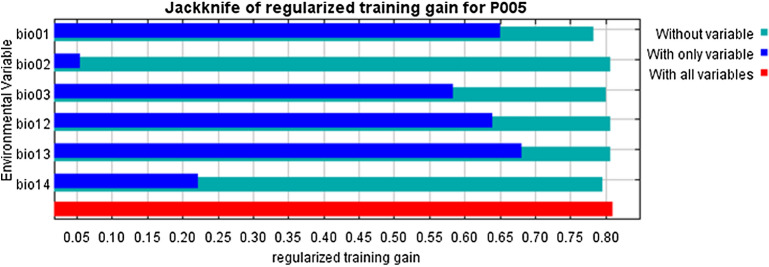
Jackknife test showing relative importance of bioclimatic variables used in RF model. The details of variables bio1, bio2, bio3, bio12, bio13, and bio 14 are presented in Table 1.

To understand the importance of the different variables in modeling the *C. odorata* distribution, we estimated the average contribution of each of the six variables over the historical data (1970–2000) are hereafter referred to as the ‘current climate’ and future (2061–2080) time periods. Among the six variables, Bio13, Bio3, and Bio1 had relatively high contributions to the model, estimated as 69.58, 23.75, and 12.59%, respectively (Table 1). This indicates a prominent role for these three variables in the distribution of *C. odorata*. The other variables had distinctly lower values and thus play more minor roles in the model. Similarly, the relative importance of each variable was assessed by Jackknife test (Fig. 1). The variables Bio13, Bio12, Bio1, and Bio3 show relatively higher importance in the model as compared to other variables (Table 2).

**Table 2:** Criteria for model evaluation and validation.

Parameter	Excellent	Good	Fair	Poor	Fail	References
AUC	0.91–1	0.81–0.90	0.71–0.80	0.61–0.7	< 0.60	^60^
TSS	0.81–1	0.80–0.61	0.41–0.60	0.40–0.21	< 0.20	^61^
Kappa	0.86–1	0.71–0.85	0.56–0.70	0.41–0.55	< 0.40	^61^

### Evaluation of RF model precision

**Figure 2:**
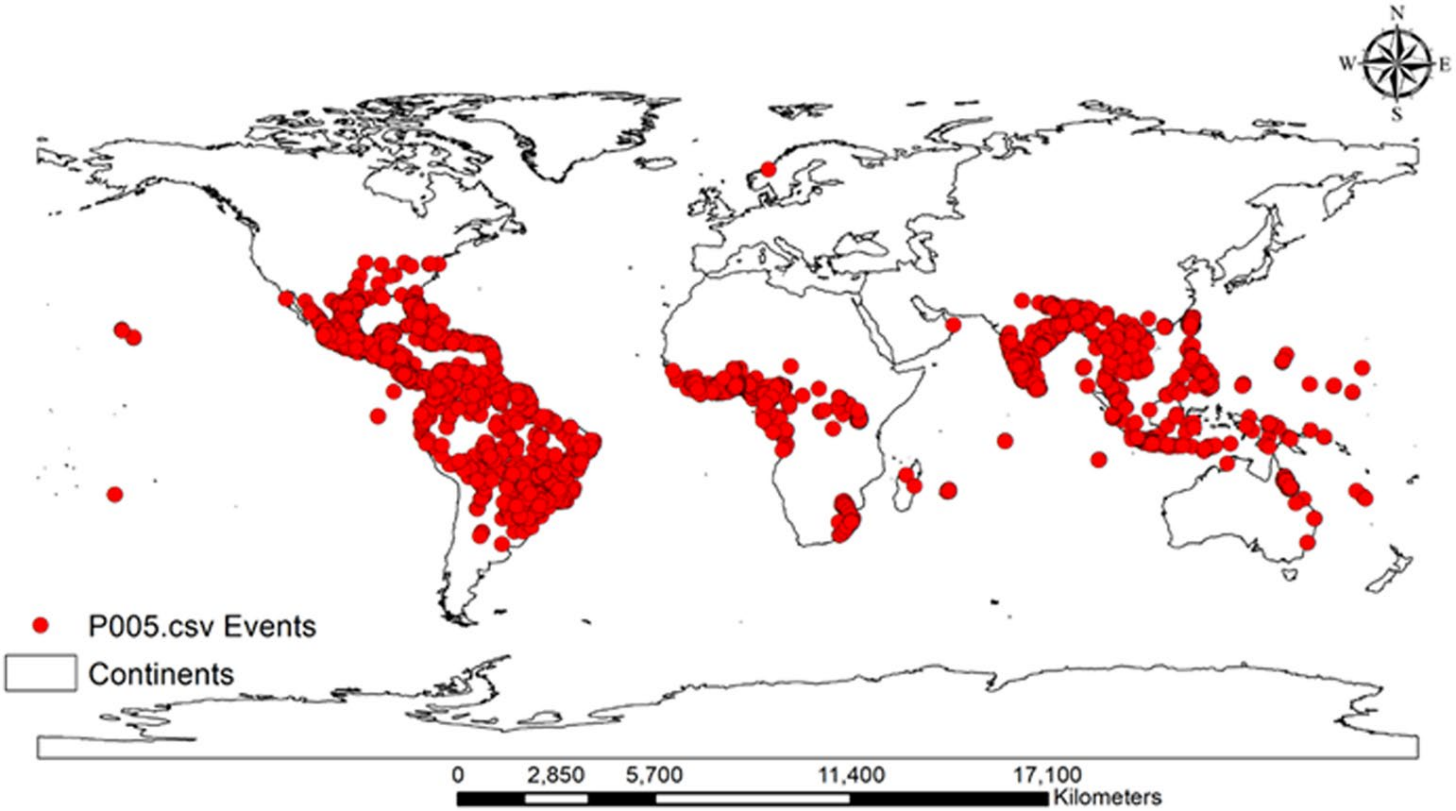
Global occurrence records of *C*. *odorata* (n = 4801). The red points in the map indicate GIS points for *C*. *odorata*. Generated using ArcGIS Desktop 10.8 (https://desktop.arcgis.com).

The area under the curve (AUC) score, true skill statistic (TSS), and Kappa coefficient were used to evaluate the performance of the model with test data. RF model performance was highest when the rarefied species-occurrence points were used rather than all occurrence points (Table 3). Therefore, we used modeling results obtained from rarefied occurrence points in this study (Fig. 2). The AUC score for the selected RF model was 0.96, indicating excellent model performance in predicting the distribution of *C*. *odorata* (Table 3). Similarly, the TSS (0.788) and Kappa coefficient (0.685) produced by the model were high. These results provide evidence that the RF model performs well and shows agreement between the observed data and predicted results.

**Table 3:** Summary of the random forest model of the worldwide distribution of *C*. *odorata*.

Model calibration	Before rarefying	After rarefying
Occurrence points	39,984	4801
AUC	0.73	0.96
TSS	0.64	0.76
Kappa	0.52	0.69

### *Spatial distribution of C*. *odorata under the current climate*

**Figure 3:**
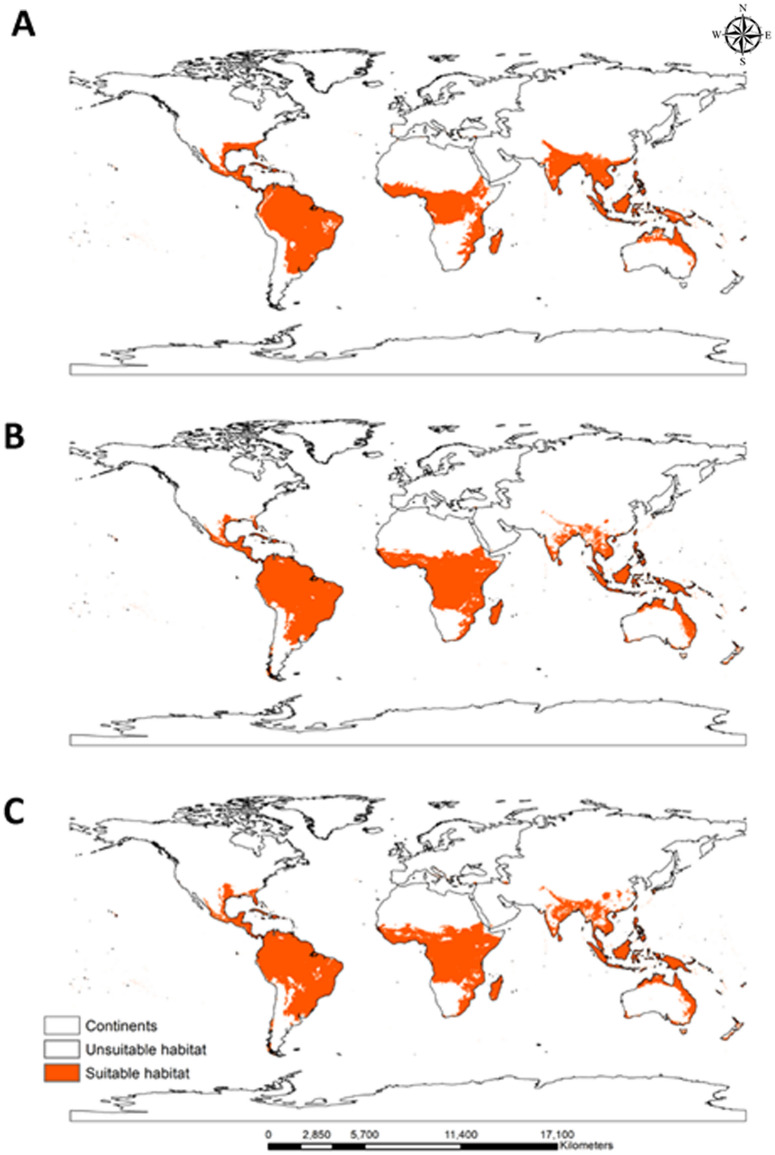
Potential global binary distribution of *C*. *odorata* under the current climate (1970–2000) (**A**) and under future climate change scenarios SSP2-4.5 (**B**) and SSP5-8.5 (**C**) (2061–2080). Orange indicates suitable habitat for *C. odorata*, and white indicates unsuitable habitat. Generated using ArcGIS Desktop 10.8 (https://desktop.arcgis.com).

RF modeling was performed to assess the current spatial distribution of *C*. *odorata* (Fig. 3A). *C. odorata is distributed at latitudes within approximately 30° north and south of the equator, although some suitable habitat areas in South America exist outside of this range.* The current spatial distribution of *C*. *odorata* was estimated in 1,753,877 cells (7,892,447 km^2^) covering 14.34% of the global land surface (Table 4). Under the current climate, *C*. *odorata* is widespread in all continents except Antarctica. The continent with the highest proportion of land within the estimated spatial distribution of *C. odorata* was South America (76.23%), followed by Africa (30.47%), Australia (21.79%), Oceania (19.95%), Asia (13.15%), North America (6.38%), and Europe (0.43%) (Table S2).

**Table 4:** Estimated habitat changes (%) from current climate to 2061–2080 for different continents under climate change scenarios SSP2-4.5 and SSP5-8.5

Continent	Total area (km^2^)	Current climate (km^2^)	Expansion (%)		Loss (%)		Unchanged (%)	
			SSP2-4.5	SSP5-8.5	SSP2-4.5	SSP5-8.5	SSP2-4.5	SSP5-8.5
Africa	6,629,265	2,020,171.5	118.67	117.41	2.70	6.02	97.30	93.98
Asia	13,730,769	1,805,130	5.73	16.74	32.02	22.76	67.98	77.24
Australia	1,798,479	391,932	85.57	81.41	28.12	29.51	71.88	70.49
Europe	3,959,239.5	17,163	5.98	44.21	86.65	59.60	13.35	40.40
North America	9,573,160.5	610,726.5	7.20	20.63	29.30	23.52	70.70	76.48
Oceania	88,200	17,595	275.65	326.04	11.99	2.33	88.01	97.67
South America	3,974,377.5	3,029,728.5	14.22	14.88	2.67	5.29	97.33	94.71
Antarctica	15,265,440	0	230	488				
Total	55,018,930.5	7,892,446.5	42.59	46.30	12.92	12.20	87.08	87.80

*SSP* shared socioeconomic pathways. Current climate, climate during 1970–2000.

### Potential changes in suitable habitat under future climate scenarios

**Figure 4:**
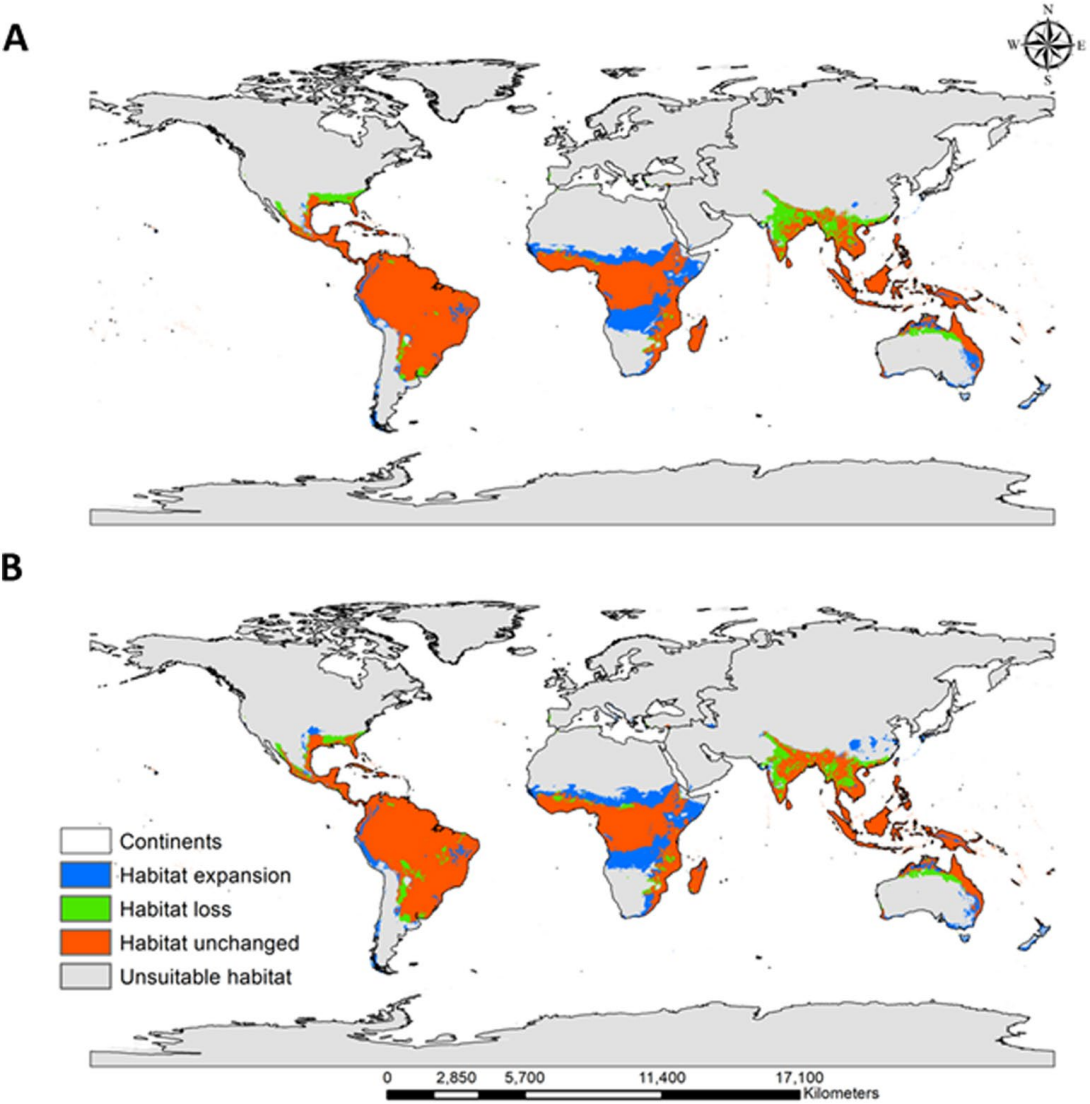
Changes in habitat suitability for *C. odorata* under the climate change scenarios SSP2-4.5 (**A**) and SSP5-8.5 (**B**) by 2061–2080. Blue indicates habitat expansion, green indicates habitat loss, orange indicates unchanged habitat, and grey indicates unsuitable habitat. Generated using ArcGIS Desktop 10.8 (https://desktop.arcgis.com).

We also used RF modeling to predict the future spatial distribution of *C. odorata* (2061–2080) under two climate change scenarios (SSP2-4.5 and SSP5-8.5; Fig. 3B and C). Overall, climate change will increase the worldwide spatial distribution of *C*. *odorata*: under SSP2-4.5 and SSP5-8.5, the area of suitable habitat for *C*. *odorata* will increase by 8.38 and 10.86%, respectively. The changes in suitable habitat for *C. odorata* (habitat expansion, habitat loss, and areas with no change) are presented in Fig. 4A (SSP2-4.5) and Fig. 4B (SSP5-8.5).

The change in suitable habitat as a proportion of current potential habitat was estimated and is expressed in Table 4. Future climate change increases the proportion of suitable habitat of *C*. *odorata* in all continents of the world, but the rate of estimated habitat expansion by 20161-2080 is the highest for Oceania (SSP2-4.5, 275.6% and SSP5-8.5, 326.1%), followed by Africa (SSP2-4.5, 118.7% and SSP5-8.5, 117.4%). Interestingly, under SSP5-8.5, even a small portion of Antarctica (0.01%) is predicted to become future potential habitat for *C*. *odorata* (SSP5-8.5). The rate of estimated habitat loss is highest for Europe (SSP2-4.5, 86.7% and SSP5-8.5, 59.6%) and lowest for South America (SSP2-4.5, 2.7% and SSP5-8.5, 59.6%) for the same future period of time. In Africa, Oceania, and South America, more than 88% of habitat is predicted to remain unchanged, but this value is less than 80% for the other continents. A tropical climate with a hot and humid environment provides a suitable habitat for *C*. *odorata*; therefore, future climate change favors the retention of current suitable habitats and further habitat expansion.

### Classification of habitat suitability in different countries of the world

**Figure 5:**
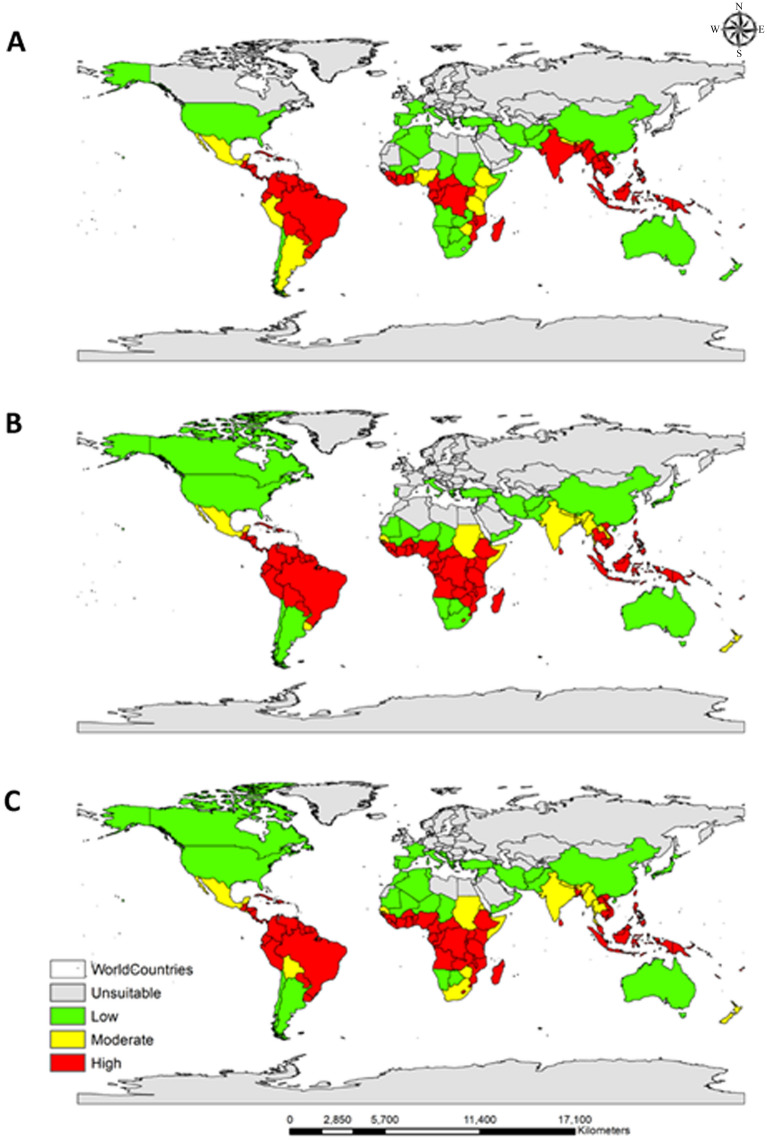
Mean habitat suitability of *C. odorata* estimated for different countries of the world. (**A**) Mean habitat suitability under the current climate. (**B** and **C**) Mean future habitat suitability under climate change scenarios SSP2-4.5 (**B**) and SSP5-8.5 (**C**) by 2061–2080. Gray indicates unsuitable habitat, green indicates habitat with low suitability, yellow indicates moderate suitability, and red indicates high suitability. Generated using ArcGIS Desktop 10.8 (https://desktop.arcgis.com).

**Figure 6:**
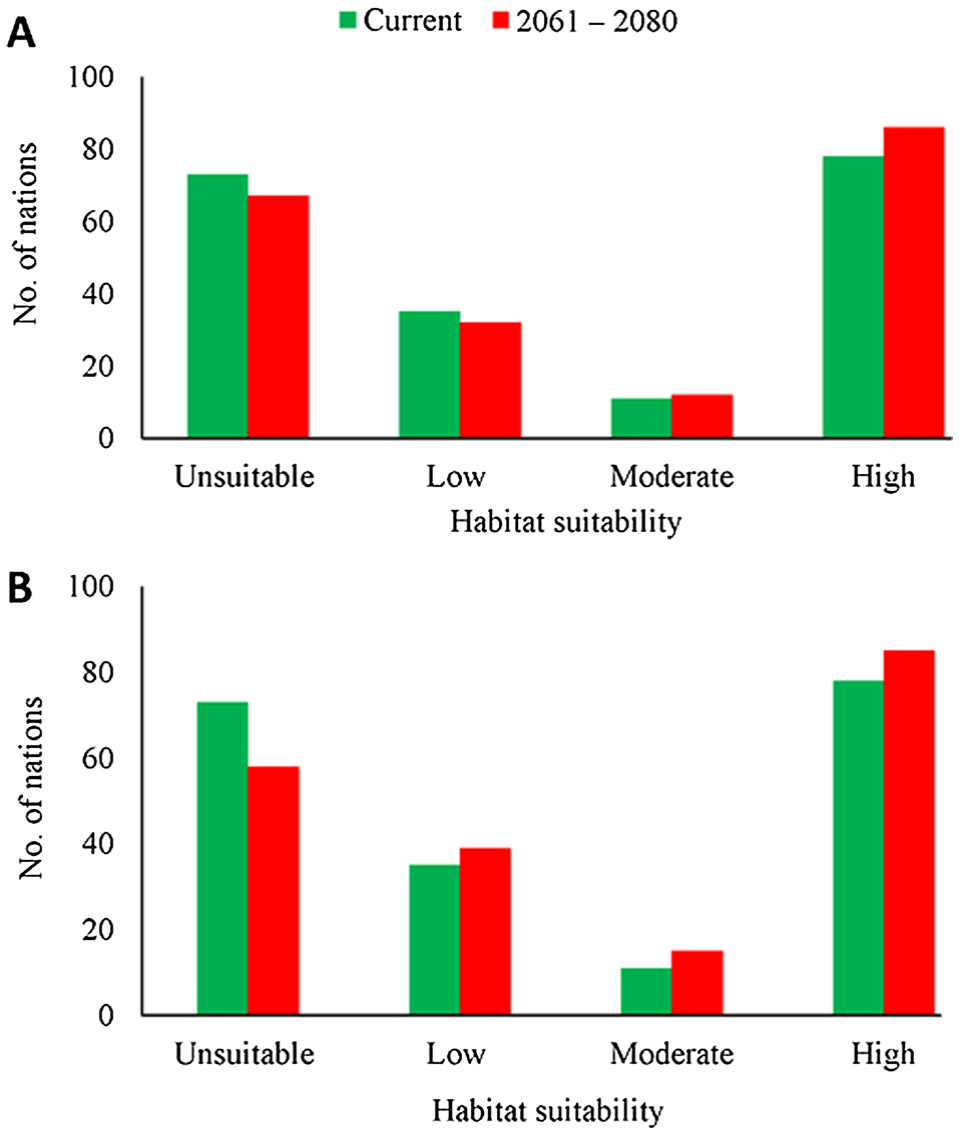
Estimation of habitat suitability in different countries of the world under the climate change scenarios SSP2-4.5 (**A**) and SSP5-8.5 (**B**). The average habitat suitability of each country was classified as unsuitable (0), low (0.1–0.33), moderate (0.34–0.66), or high (0.67–1.00). Green indicates the number of countries currently in each category; red indicates the predicted number of countries in each category in 2061–2080.

The mean habitat suitability of *C. odorata* was estimated for all countries (Table S3). Each country was subsequently classified as being unsuitable or having low, moderate, or high suitability for *C. odorata* under the current climate (Fig. 5A) and the two climate change scenarios (SSP2-4.5, Fig. 5B; SSP5-8.5, Fig. 5C*).* Under the current climate, 73 countries are classed as having unsuitable habitats (Fig. 6). However, under SSP2-4.5, by 2061–2080, 9 of these 73 countries are predicted to transition to the low-suitability category (Canada, Cape Verde, Djibouti, Japan, Mauritania, Niger, Norway, Yemen), 1 to the moderately suitable category (Senegal), and 3 to the highly suitable category (Gambia, Guinea-Bissau, and Lesotho) (Tables 5 and S4).

**Table 5:** Predicted *C. odorata* habitat transition in different countries from current climate to 2061*–*2080 under climate change scenarios SSP2-4.5 and SSP5-8.5

Change in habitat suitability^a^	SSP2-4.5	SSP5-8.5
Unsuitable to low	Canada, Cape Verde, Djibouti, Japan, Mauritania, Niger, Norway, Yemen	Bosnia and Herzegovina, Canada, Croatia, Djibouti, Israel, Japan, Mauritania, Montenegro, Niger, South Korea, Yemen
Unsuitable to moderate	Senegal	Senegal
Unsuitable to high	Gambia, Guinea-Bissau, Lesotho	Gambia, Guinea-Bissau, Lesotho
Low to moderate	Eritrea, New Zealand, Somalia, Sudan	Eritrea, New Zealand, Somalia, Sudan
Low to high	Angola, Burkina Faso, Zambia	Angola, Burkina Faso, Zambia
Moderate to high	Benin, Ethiopia, Nigeria, Peru, Tanzania, Zimbabwe	Benin, Ethiopia, Kenya, Nigeria, Peru, Tanzania

^a^The habitat suitability categories were unsuitable (0), low suitability (0.001–0.33), moderate suitability (0.34–0.66), and high suitability (0.67–10), and categorization was performed according to the mean habitat-suitability value for each country. This table shows that some countries in the unsuitable, low suitability, and moderate-suitability categories in current climate are predicted to transition to a higher level of habitat suitability by 2061*–*2080 under climate change scenarios SSP2-4.5 and SSP5-8.5. Details of habitat suitability classification are presented in Table S4.

Similar results are predicted under SSP5-8.5 but with additional countries, including South Korea, Bosnia and Herzegovina, Israel, and Montenegro, predicted to transition from unsuitable to low suitability (Table 5). Eritrea, New Zealand, Somalia, and Sudan are predicted to transition from low to moderate suitability under SSP5-8.5, and Angola, Burkina Faso, and Zambia are predicted to transition from low to high suitability. The South American and African countries up to 30°S are predicted to have moderate to high suitability, but the Asian and European countriesabove 30°N latitude are predicted to have unsuitable to low-suitability habitats. These results indicate that several countries in Africa and South America, and some countries in Asia, may be at high risk of invasion by *C. odorata* in the future.

## Discussion

The key findings of this study are as follows. First, the model predictions from rare species-occurrence points are more accurate and less likely to cause overestimation with the RF model (Table 3). Second, the current global spatial distribution of *C*. *odorata* is primarily concentrated in South America and Africa, with minor regions in Asia and Australia (Fig. 3A). In general, suitable habitats are predominantly located between 30° north and south of the equator. However, future climate change will allow the spread of suitable habitats across all continents, with Oceania experiencing significant habitat expansion (326.04%) relative to its current status. Third, under current climate conditions, 73 countries have habitats that are not suitable for *C*. *odorata*. Of these countries, 11 (Bosnia and Herzegovina, Canada, Croatia, Djibouti, Israel, Japan, Mauritania, Montenegro, Niger, South Korea, and Yemen) will change to low suitability, 1 (Senegal) will change to moderate suitability, and 3 (Gambia, Guinea-Bissau, and Lesotho) will change to high suitability under the SSP5-8.5 scenario (Table 5). Last, tropical and warm temperate climates are favorable for *C*. *odorata*; therefore, northern Asia, including Mongolia, Kazakhstan, and Russia, and central and northern Europe are predicted to be safe from invasion of *C*. *odorata* for the studied time period (2061–2080; Fig. 3B and C).

Species distribution models (SDMs) are commonly used to predict the potential risk of invasion by alien species^28^. The accuracy of these models depends on factors such as the algorithms and variables used^29^, and the quality and quantity of the species-occurrence data^30^. The latter is particularly important for proper modeling. The species occurrence data used in this study was from GBIF. The GBIF data sources may have taxonomic bias and this bias can arise from a variety of factors, including variation in the ease of identification, the frequency of occurrence, sampling protocol, quality control procedures and the level of interest among the researchers or citizen scientist, and manual georeferencing of herbarium or museum specimens^31^. Therefore, we carefully check the taxonomy, data source, and implemented some quality measures such as filters to remove occurrences with improbable geographic coordinates. During the process we removed the occurrence points present in sea, desert and in arctic regions.

In this study, we conducted RF modeling both with all available occurrence points and with spatially rarefied occurrence points, to reduce errors and overfitting due to spatial autocorrelation^32^. We compared model performance using the AUC scores, TSS, and Kappa coefficients (Table 3): the model that used the spatially rarefied occurrence points produced the highest scores and was thus considered the most accurate. Our RF model makes accurate predictions and can simulate the spread of *C. odorata*, but there are limitations to this study. The assumption that species niche demand is conservative may not always be true as invasive species can experience niche drift^33^. Additionally, invasive species often move quickly to many places, making it difficult to accurately predict their potential distribution^34^. Using equilibrium data to identify suitable habitat areas would be more effective, but monitoring the population status of invasive species in invaded areas is currently difficult and a low priority. These issues are similar to the limitations reported in studies of *Xanthium italicus*^34^.

The distribution and modeling of invasive species can be influenced by both intrinsic factors, such as dispersal distance and rate of species generation, and extrinsic factors, such as human activities and natural phenomena^35^. According to the Jackknife test performed in this study, two temperature-related variables Bio 1 and Bio 3 and two precipitation-related variables Bio 12 and Bio 13 and were relatively important variables for the habitat suitability of *C*. *odorata* (Fig. 1), estimated to contribute 12.59, 23.75, 2.40 and 69.58%, respectively (Table 1). *C*. *odorata* thrives in areas with high temperatures and precipitation but is unable to tolerate frost^9^. The seeds of *C*. *odorata* are susceptible to damage at extremely cold temperatures and in excessively dry soil^9^. As a result, it prefers wet-dry seasonal conditions and can grow in various soils up to 1200 m above sea level. Therefore, future climate change is expected to promote the habitat expansion of *C*. *odorata’s* habitat^36^.

Besides bioclimatic variables, several traits contribute to the invasive nature of *C. odorata*, including its high germination and growth rate, high fecundity^37^, ability to regenerate from roots, and ability to tolerate a wide range of temperatures^10, 38^. Environmental factors such as anthropogenic land-use and landcover changes, soil, and roads, may also be crucial factors in determining the distribution of *C. odorata*, but future data on such variables are not yet accessible at a similar resolution. The plant can spread easily via wind, fur, clothes, and machinery and can quickly take over new habitats once introduced, replacing other invasive species^38^. Studies have found that abandoned farms, orchards, urban environments, and roadside ditches make excellent habitats for *C. odorata*, allowing it to rapidly dominate these areas^9, 10^.

Our study shows that *C. odorata* is primarily found in areas between 30° north and south of the equator, with humid tropical, subtropical, and warm temperate climates. We also found that the greatest habitat expansion for *C*. *odorata* is predicted to occur in Africa, specifically in Nigeria, Chad, Sudan, and Somalia. *C. odorata* is also expected to expand in North America, South America, Asia, Australia, and New Zealand, resulting in an increase in its spatial distribution of up to 10.86%. An increase in temperature and precipitation patterns may lead to the future loss of habitat for native species and the transition of habitat from unsuitable for *C. odorata* to highly suitable for *C. odorata*, with its invasiveness aided by its high reproductive capacity, similar to other invasive plants such as *P. hysterophorus*^26^. However, Mediterranean, semiarid, and cold temperate climates are predicted to be unsuitable habitats for *C. odorata* in the future. Most parts of North America, Europe, and northern and central Asia are also predicted to be unsuitable habitats for the plant. These results are consistent with some previous studies^9, 10, 38^.

The Antarctic Peninsula has experienced the most rapid air-temperature increases in the world over the past 50 years, and the West Antarctic Ice Sheet is projected to lose nearly all of its bulk over several millennia, with sustained warming levels between 2 and 3 °C^39^. These changes could result in the loss of native biodiversity and the establishment of alien and invasive species in Antarctica. Although the northern hemisphere above 30°N latitude is currently unsuitable for *C*. *odorata*, we found that a small portion of Antarctica (0.01%) could potentially become a suitable habitat for this invasive species in the future because of climate change. Therefore, further research is needed to investigate the potential for *C*. *odorata* to establish in Antarctica.

*C. odorata* grows rapidly and can negatively impact agriculture, forestry, and grazing animals^40^. Its leaves, particularly the young ones, contain high levels of nitrate, making them toxic and sometimes fatal to grazing cattle and wild herbivores such as roe deer^41^. *C. odorata* is difficult to control once established as it can spread quickly and regrow from seeds and rootstocks, even in hard-to-reach places like cliffs^40^. It also alters soil nutrients and affects nearby vegetation^5^ and is named in “100 of the World’s Worst Invasive Alien Species”^4^. Despite these negative impacts, practitioners of conventional medicine continue to use it for its various medicinal properties and bioactive compounds, such as fatty acids, flavonoids, saponins, and alkaloids^42^.

*C. odorata* is an invasive plant species that requires a combination of strategies to control its spread and mitigate its negative impacts on the ecosystem, including herbicides, mechanical control, and biological control^43^. Herbicides can be effective but also harm insects and native flora. Alternative control methods, such as mechanical or biological controls, have fewer side effects but can be costly and require more effort. Biological methods of control have been used in many countries with success rates of up to 70%^44^. A strict quarantine system on borders is needed to stop the further expansion of *C. Odorata*; collaboration between governments, land-resource managers, and local stakeholders will be required to develop an invasion control plan.

## Conclusions

*C*. *odorata* can adapt to changing climate conditions such as rising temperatures and shifting precipitation patterns. In this study, an RF model was used to estimate the global spatial distribution of *C*. *odorata* and to identify areas at high risk of invasion under current and future climate scenarios (SSP2-4.5 and SSP5-8.5). The results revealed that, by 2016–2080, *C*. *odorata* will retain its current ecological niche and expand its habitat in many countries of Oceania, Africa, and South America, with expansion of up to 326.04% in Oceania. The global distribution of *C*. *odorata* is expected to increase by up to 10.86% under the SSP5-8.5 climate change scenario. Countries with tropical, subtropical, and temperate climates located between 30° north and south of the equator are at high risk of invasion. Our study identified areas that may become suitable habitats under current and future climate change, which will be useful in developing long-term management strategies for *C. odorata*. For examples, immediate control and management measures such as mechanical control, biological control, and restoration of native species are needed in high-risk countries. These countries should establish the preventive measures such as robust quarantine systems against a potential threat of invasion.

## Materials and methods

### Global species-occurrence records

Global occurrence records for *C*. *odorata* (39,984 points) were downloaded mainly from the GBIF (www.gbif.org, accessed September 8, 2022), an open access data source. The spatially rarefy occurrence tool in the ArcGIS SDM toolbox v. 2.4 was then used to remove multiple species-occurrence points from the same grid at a spatial scale of 2.5 min (4.5 km^2^) and to select one distinct point per grid^45^. This procedure prevents overfitting and inaccurate inflation of model results due to spatial autocorrelation^32^. Ultimately, there were 4,801 species-occurrence points for *C*. *odorata* (Fig. 2 and Table S5). In this study, we used both sets of species-occurrence points in the RF modeling of *C*. *odorata* to understand model overestimation.

### Selection of bioclimatic variables

Temperature and precipitation are two of the most significant environmental variables that determine floral diversity and distribution. Thus, the 19 WorldClim bioclimatic variables (derived from temperature and precipitation data) are highly relevant to the ecological and physical tolerance of plants^46^. We therefore downloaded historical data from 1970 to 2000 from WorldClim v2.1 at a spatial resolution of 2.5 min, which is approximately 4.5 km at the equator^47^. Similarly, future bioclimatic variables at 2.5 min resolution from the Coupled Model Intercomparison Project Phase 6 (CMIP6) were also downloaded from the WorldClim data portal^48^. We also used two shared socioeconomic pathways (SSPs; SSP2-4.5 and SSP5-8.5) representing future climate data from 2061 to 2080, developed under the global circulation model (GCM) Max Planck Institute Earth System Model (MPI-ESM1-2-HR)^49^. The SSP scenarios assess changes in energy use and land use as well as the associated uncertainties in greenhouse gas and air pollutant emissions^50^. Among the four SSP scenarios in the WorldClim database, the SSP2-4.5 and SSP5-8.5 depicted an average warming of 3.0 and 5.0 °C, respectively. These scenarios represented intermediate and high radiative forcing, which encompassed greenhouse gas emissions, forest fires, and volcanic eruptions^48^. Consequently, the modeling of *C. odorata* utilized the SSP2-4.5 and SSP5-8.5 scenarios.

According to SSP2-4.5 and SSP5-8.5, the average global surface temperature in 2090–2100 will have risen by 2.4–4.3 °C and 3.8–8.6 °C, respectively, relative to 1880–1900^48^. The SSPs anticipate worldwide socioeconomic development through 2100. The MPI-ESM1-2-HR GCM includes land and ocean carbon cycles, and encompasses the latest ocean biogeochemistry module (the Hamburg Model of the Ocean Carbon Cycle) and land surface scheme (JSBACH)^51^ to predict the seasonal climate over decades. Downloading bioclimatic variables from the WorldClim data portal is a popular method of estimating the probable spread of species in response to changing precipitation and temperature. Depending on the particular climate scenarios and associated ecologies, the bioclimatic variables serve to define and forecast the future distribution patterns of species^34^.

We ran a Spearman’s correlation test on data from the 19 bioclimatic WorldClim variables (Table S6) using the PROC CORR function of SAS 9.4 (SAS Institute, Inc., Cary, NC, USA), as described before^52, 53^. We selected 6 of the 19 bioclimatic variables (Table 1) on the basis of their low correlation with each other (r < 0.75; Table S1): annual mean temperature (Bio01), mean diurnal temperature range (Bio2), isothermality (Bio03), annual precipitation (Bio12), precipitation in the wettest month (Bio13), and precipitation in the driest month (Bio14). These six variables were considered the most significant climatic variables for predicting the spatial distribution of *C. odorata.* The relative importance of each variable was accessed using Jackknife test.

### Model development

The RF model predictions for *C*. *odorata* in this study were performed with Biomod2 Package 4.1-2, selecting single model RF^54^. The species-occurrence data and bioclimatic data were imported into R. The lack of information about invasive species can make it difficult to determine whether a habitat is suitable because invasive species are likely expanding and have not yet reached equilibrium^55^. The background data (pseudo absent) points of the study area were determined using ArcGIS 10.3, as suggested previously^56, 57^. The species-occurrence data were divided into two; 75% of the data was used for model calibration, and 25% was used for model validation^58^. The other model options were run with the default settings, and the model was replicated ten times, as described previously^59^.

### Model evaluation and validation

The goodness-of-fit of the model was evaluated via three parameters, namely, the AUC score of the receiver operating characteristic (ROC) curves^60^, the TSS^61^, and the Kappa coefficient. The AUC score plots 1 specificity on the abscissa and sensitivity on the ordinate^14^. The AUC is a technique for differentiating between presence and absence that is independent of thresholds, and the score, which assesses the performance of a model, ranges from 0 to 1^62^. The AUC value is unaffected by the size of the dataset (prevalence), but its use is debatable because it gives equal weight to errors of commission and omission and may not assess prediction accuracy reliably^63^. Habitat expansion outside of the species-occurrence range may provide a high AUC score, leading to overfitting, a condition that misleads model evaluation^64^. Therefore, other evaluation parameters (TSS and Kappa coefficient) were also employed to measure the accuracy of the model. The TSS calculates both the specificity and sensitivity of the model [TSS = sensitivity + (specificity − 1)]^61^, ranging from − 1 to + 1; estimates both omission and commission errors^61^; and is frequently used as an alternative method for checking model accuracy^61, 65^. The Kappa coefficient is the ratio of observation points that the model properly and erroneously predicts. Similar to the TSS, it also ranges from − 1 (poor agreement) to + 1 (perfect prediction)^61, 66^. The greater the scores of these parameters, the higher the accuracy of the model (Table 2)^67^.

### *Spatial distribution and change in habitat suitability of C*. *odorata across the world*

The global binary distribution maps of *C*. *odorata* were obtained from probability distribution maps produced by RF modeling under the current and future climate change scenarios (SSP2-4.5 and SSP5-8.5) for the period 2061–2080 using the threshold TSS defined in the Biomod2 Package^54^. The binary distribution maps represent the suitable and unsuitable habitats for *C*. *odorata.* We estimated the changes in suitable habitats for *C*. *odorata* by 2061–2080 by differentiating current suitable and future suitable habitats, and reclassifying the differentiated raster to identify expanded, reduced, and unchanged regions across the world using the Raster v3.6 package in R software (https://cran.r-project.org/web/packages/raster, accessed December 12, 2023). The number of cells showing suitable habitat under the current climate and the number of expanded, reduced, and unchanged habitats by 2061–2080 were estimated for the different continents using zonal statistics under the spatial analyst in ArcGIS Desktop 10.8 (Esri, Redlands, CA, USA). Then, the approximate area was estimated (1 grid cell = 4.5 km^2^), and the proportions of habitat change for the different continents were calculated under the climate change scenarios SSP2-4.5 and SSP5-8.5. Similarly, to understand the risk of invasion in different countries of the world, mean habitat-suitability values for 2061–2080 were estimated for countries under the current and future climate change scenarios, and countries were then classified into four categories: unsuitable habitat (0), low-suitability habitat (≤ 0.33), moderate-suitability habitat (≤ 0.66), and high-suitability habitat (≤ 1).

## Supplementary Information

Supplementary Information 1.

Supplementary Information 2.

Supplementary Information 3.

Supplementary Information 4.

Supplementary Information 5.

Supplementary Information 6.

## Data Availability

All data generated or analyzed during this study are included in this article.
